# CHALLENGES TO UNDERGRADUATE AND GRADUATE PROGRAMS AT THE UNIVERSITY OF SOUTH FLORIDA

**DOI:** 10.1093/geroni/igad104.0636

**Published:** 2023-12-21

**Authors:** Debra Dobbs, William Haley, Nasreen Sadeq

**Affiliations:** University of South Florida, Tampa, Florida, United States; University of South Florida, Tampa, Florida, United States; University of South Florida, Tampa, Florida, United States

## Abstract

The University of South Florida has more than 50,000 students. The School of Aging Studies first offered an MA in Gerontology in 1967. We currently offer many undergraduate General Education courses, two undergraduate majors, online graduate certificate and master’s programs, and a PhD in Aging Studies. Accountability and addressing university metrics and priorities have become an increasing requirement at USF and elsewhere. We have had great success in our General Education courses, consistently reaching over 5,000 students per year and producing high Student Credit Hour (SCH) numbers, an important metric. Recently, our programs experienced challenges with increased class sizes resulting from higher demand for our general education courses. In several cases, the number of course sections offered and the number of seats in each had doubled. Meeting these demands placed a strain on faculty, instructors, and graduate student assistants. We also face challenges as our university funding formula increasingly focuses on undergraduate major enrollment, and therefore we are focusing on innovative methods to recruit more students at the undergraduate, master’s, and PhD level. In this presentation, we will describe some of the strategies that have maximized our successes, and efforts to address challenges in sustaining larger numbers of degree seeking students in our programs. We will include data on SCH, grant funding, publications, citations, and program graduates per year which help us monitor our successes and areas needing improvement.

